# Plasma interferon-alpha is associated with double-positivity for autoantibodies but is not a predictor of remission in early rheumatoid arthritis—a spin-off study of the NORD-STAR randomized clinical trial

**DOI:** 10.1186/s13075-021-02556-1

**Published:** 2021-07-13

**Authors:** Marit Stockfelt, Anna-Carin Lundell, Merete Lund Hetland, Mikkel Østergaard, Till Uhlig, Marte Schrumpf Heiberg, Espen A. Haavardsholm, Michael T. Nurmohamed, Jon Lampa, Dan Nordström, Kim Hørslev Petersen, Bjorn Gudbjornsson, Gerdur Gröndal, Jonathan Aldridge, Kerstin Andersson, Kaj Blennow, Henrik Zetterberg, Ronald van Vollenhoven, Anna Rudin

**Affiliations:** 1 Department of Rheumatology and Inflammation Research, Institute of Medicine, Sahlgrenska Academy at the University of Gothenburg, Guldhedsgatan 10A, S-405 30 Gothenburg, Sweden; 2 Rheumatology Clinic, Sahlgrenska University Hospital, Gothenburg, Sweden; 3 Copenhagen Center for Arthritis Research, Center for Rheumatology and Spine Diseases, Rigshospitalet, Glostrup, Denmark; 4 Department of Clinical Medicine, Faculty of Health and Medical Sciences, University of Copenhagen, Copenhagen, Denmark; 5 Division of Rheumatology and Research, Diakonhjemmet Hospital, Oslo, Norway; 6 Institute of Clinical Medicine, University of Oslo, Oslo, Norway; 7 Amsterdam Rheumatology and Immunology Center, Reade, Amsterdam, The Netherlands; 8 Department of Rheumatology and Amsterdam Rheumatology Center, Amsterdam University Medical Centres, Amsterdam, The Netherlands; 9 Department of Medicine, Rheumatology Unit, Center for Molecular Medicine (CMM), Karolinska Institute, Karolinska University Hospital, Stockholm, Sweden; 10 Department of Medicine and Rheumatology, Helsinki University and University Hospital, Helsinki, Finland; 11 Danish Hospital for Rheumatic Diseases, University Hospital of Southern Denmark, Sønderborg, Denmark; 12 Department of Regional Health Research, University of Southern Denmark, Odense, Denmark; 13 Centre for Rheumatology Research, Landspitali University Hospital, Reykjavik, Iceland; 14 Faculty of Medicine, University of Iceland, Reykjavik, Iceland; 15 Department of Psychiatry and Neurochemistry, Institute of Neuroscience and Physiology, Sahlgrenska Academy at the University of Gothenburg, Gothenburg, Sweden; 16 Clinical Neurochemistry Laboratory, Sahlgrenska University Hospital, Mölndal, Sweden; 17 UK Dementia Research Institute at UCL, London, UK; 18 Department of Neurodegenerative Disease, UCL Institute of Neurology, London, UK

## Abstract

**Background:**

The type I interferon (IFN) gene signature is present in a subgroup of patients with early rheumatoid arthritis (RA). Protein levels of IFNα have not been measured in RA and it is unknown whether they associate with clinical characteristics or treatment effect.

**Methods:**

Patients with early untreated RA (n = 347) were randomized to methotrexate combined with prednisone, certolizumab-pegol, abatacept, or tocilizumab. Plasma IFNα protein levels were determined by single molecular array (Simoa) before and 24 weeks after treatment initiation and were related to demographic and clinical factors including clinical disease activity index, disease activity score in 28 joints, swollen and tender joint counts, and patient global assessment.

**Results:**

IFNα protein positivity was found in 26% of the patients, and of these, 92% were double-positive for rheumatoid factor (RF) and anti-citrullinated protein antibodies (ACPA). IFNα protein levels were reduced 24 weeks after treatment initiation, and the absolute change was similar irrespective of treatment. IFNα protein positivity was associated neither with disease activity nor with achievement of CDAI remission 24 weeks after randomization.

**Conclusion:**

IFNα protein positivity is present in a subgroup of patients with early RA and associates with double-positivity for autoantibodies but not with disease activity. Pre-treatment IFNα positivity did not predict remission in any of the treatment arms, suggesting that the IFNα system is distinct from the pathways of TNF, IL-6, and T-cell activation in early RA.

A spin-off study of the NORD-STAR randomized clinical trial, NCT01491815 (ClinicalTrials), registered 12/08/2011, https://clinicaltrials.gov/ct2/show/NCT01491815.

**Supplementary Information:**

The online version contains supplementary material available at 10.1186/s13075-021-02556-1.

## Introduction

Rheumatoid arthritis (RA) is a chronic disease characterized by joint inflammation, which if untreated may lead to progressive bone destruction. Genetic and environmental factors contribute to the predisposition towards disease development, including smoking and genes of the type I interferon (IFN) pathway [1–3]. The majority of patients with RA have autoantibodies against the Fc portion of IgG (rheumatoid factor (RF)) and/or citrullinated peptides (ACPA). Two studies have shown that ACPA positivity is associated with elevated expression of type I IFN responsive genes (IRG) in RA [4, 5], while others have reported that these factors are unrelated [6, 7]. Whether RF or ACPA are associated with IFNα protein is unknown.

The majority of IFNα is produced by plasmacytoid dendritic cells following their recognition of microbial nucleic acids and immune complexes. Binding to the type I IFN receptor leads to upregulation of genes involved in immune processes including restriction of viral replication and enhancement of B cell responses [8]. A persistent upregulation of IRG, the type I IFN signature, is evident in several autoimmune diseases including systemic lupus erythematosus (SLE) and RA [9]. In RA, the expression of IRG is upregulated in peripheral blood compared to controls [10] and was suggested to associate with disease activity [11] and predict treatment response to tumor necrosis factor inhibitors (TNFi) [12–14], interleukin-6 receptor inhibitors (IL-6Ri) [15], and B-cell depletion therapy [16–19]. However, the stimulation of IRG expression is not specific for IFNα and which genes to include is not standardized. Since functional bioassays are not specific for IFNα, and traditional ELISAs are insufficiently sensitive, a reliable method to measure IFNα protein has been lacking. Recently, a digital ELISA based on single molecular array (Simoa) was developed that enables direct quantification of IFNα at attomolar levels [20]. In SLE, IFNα protein associated with disease activity and predicted the duration of remission [21], but protein levels of IFNα have previously neither been reliably measured in RA nor related to clinical characteristics or treatment effect.

Early and effective medical treatment improves well-being and prognosis in RA. Current European and US guidelines advocate initiating treatment with methotrexate (MTX) or other conventional synthetic disease-modifying anti-rheumatic drug (DMARD) [22, 23]. If the therapeutic effect is insufficient, another conventional, biologic, or targeted synthetic DMARD may be added. In the NORD-STAR cohort, active conventional treatment and biologic treatment with certolizumab-pegol, abatacept, and tocilizumab were compared head-to-head [24]. All four treatments achieved high remission rates on a group level. At the individual level, it may be possible to predict treatment effect using biomarkers, but specific biomarkers that inform on the effect of different treatment strategies in early RA are lacking.

We used plasma samples from the Swedish patients in the NORD-STAR cohort to explore whether IFNα protein positivity is present in patients with early untreated RA, whether levels of IFNα change after treatment with conventional and biologic treatment strategies, and whether baseline IFNα protein levels predict remission at week 24.

## Materials and methods

### Study population

The study population consisted of 347 Swedish patients included in the NORD-STAR trial, a multinational phase four, investigator-initiated, randomized observer-blinded clinical trial of 812 patients with early untreated RA [24]. All patients fulfilled the American College of Rheumatology (ACR) and European League Against Rheumatism (EULAR) 2010 criteria. Patients were assessed for eligibility during 2012–2018. All patients were of age 18 or above, had a symptom duration of fewer than 24 months, and at least two (of 66) swollen and two (of 68) tender joints. All patients had to be RF and/or ACPA positive or have a C-reactive protein (CRP) of at least 10 mg/L. All patients had moderate to severe disease activity score (DAS28-CRP ≥ 3.2) and all were DMARD naïve. Active infection or any major episode of infection requiring hospitalization within 4 weeks of screening constituted exclusion criteria. All participants signed a written informed consent and the study was approved by the regional ethics board in Stockholm (d.nr. 2011/2069-31/4 and amendment 2019-05705).

### Intervention

Details of the study protocol and data regarding clinical outcome at week 24 in the full NORD-STAR cohort are published [24, 25]. In brief, Swedish patients were randomized 1:1:1:1 stratified by ACPA and sex to MTX escalated to 25 mg/week with folic acid supplementation combined with one of the following: arm 1, active conventional treatment (oral prednisone tapered from 20 to 5 mg/day in 9 weeks); arm 2, TNFi (certolizumab-pegol, 200 mg subcutaneously every other week, loading dose 400 mg at weeks 0, 2, and 4); arm 3, cytotoxic T-lymphocyte-associated molecule-4 immunoglobulin (CTLA-4Ig, abatacept, 125 mg subcutaneously every week); or arm 4, IL-6Ri (tocilizumab, 8 mg/kg intravenously every 4 weeks or 162 mg subcutaneously every week). There was no difference between the intention-to-treat and the per-protocol treatment arm. Oral steroids were not allowed for patients who received a biological DMARD (arm 2–4). Intra-articular corticosteroid injections were allowed on demand up to week 20 in arm 1 and until week 12 in arm 2–4. If an oral dose of 25 mg/week MTX was not tolerated, the dose was reduced or changed to subcutaneously administered MTX; if MTX was still not tolerated, it was replaced with leflunomide or azathioprine, or monotherapy for patients on biologic medication. None of the patients was treated with hydroxychloroquine.

### Clinical evaluation

The primary clinical endpoint was remission according to the clinical disease activity index (CDAI ≤ 2.8) at week 24. In addition, disease activity was evaluated on day 1 before the start of treatment and 24 weeks after treatment initiation with the following parameters: CRP, erythrocyte sedimentation rate (ESR), DAS28-ESR and DAS28-CRP, swollen joint count in 66 joints (SJC66), tender joint count in 68 joints (TJC68), and patient global assessment (PGA). Positivity for ACPA and RF was determined according to cut-off levels at the local laboratories.

### Quantification of IFNα in plasma

Plasma was kept frozen until analysis. Plasma IFNα protein concentration was measured with Simoa on an HD-1 Analyzer (Quanterix, Billerica, MA). The analysis was performed blinded to patient characteristics. The Simoa assay contained an inhibitor for RF and heterophilic antibodies in order to prevent false-positive results. Values below the detection limit were assigned the lowest limit of detection (LLOD, 70 fg/mL). Within-run and between-run coefficients of variation (CVs) for the Simoa assay were 9.8% and 7.3% at 1.9 pg/mL and 8.1% and 7.3% at 10.6 pg/mL. The assay was not controlled for concentrations lower than 1.9 pg/mL. IFNα protein positivity was defined as an IFNα level ≥ 136 fg/mL, based on three standard deviations above mean level for healthy blood donors, measured using the same method [21]. IFNα protein levels could not be obtained due to a technical error in one sample collected at baseline and one sample collected at 24 weeks.

### Statistics

Mann-Whitney U-test, Wilcoxon matched-pairs signed rank test, Kruskal-Wallis test followed by Dunn’s multiple comparison test (GraphPad Prism software v9.02, La Jolla, CA), and Fisher’s exact test (IBM SPSS Statistics v27, Armonk, NY) were used as described in the respective figure legends. For analysis of autoantibody status in relation to IFNα, after Fisher’s exact test, a post hoc step-down Bonferroni-Holm correction for multiple testing was performed. Multivariable logistic regression was used to identify factors independently associated with IFNα protein positivity and identify whether IFNα protein positivity was independently associated with remission at week 24 (GraphPad Prism software). A p-value of < 0.05 was considered statistically significant (*P < 0.05, **P < 0.01, ***P < 0.001, and ****P < 0.0001).

## Results

### IFNα protein positivity is present in a subgroup of untreated early RA patients

**Fig. 1:**
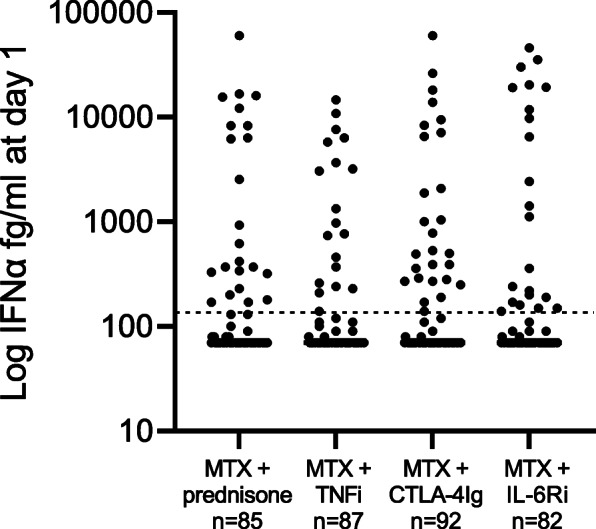
Elevated IFNα protein levels at baseline in early RA. IFNα protein levels in plasma from patients with early RA before treatment initiation in four treatment arms, methotrexate + prednisone, methotrexate + TNFi, methotrexate + CTLA-4Ig, and methotrexate + IL-6Ri. The dotted line denotes the cut-off for IFNα positivity (136 fg/mL). MTX (methotrexate), TNFi (certolizumab-pegol), CTLA-4Ig (abatacept), and IL-6Ri (tocilizumab). Kruskal-Wallis test followed by Dunn’s multiple comparison test

Baseline demographic and clinical characteristics of the 347 patients with untreated early RA in each treatment arm are shown in Table 1. There were no significant differences in baseline characteristics between the four treatment arms. Of the 346 patients with data for plasma IFNα protein levels at baseline, 26% (n = 91) were IFNα-positive, with similar proportions in the four treatment arms, i.e., methotrexate in combination with either prednisone (27%, n = 23), TNFi (22%, n = 19), CTLA-4Ig (29%, n = 27), or IL-6Ri (27%, n = 22) (Fig. 1).

**Table 1:** Baseline characteristics of untreated patients with early RA in the four treatment arms

N = 347	MTX + prednisone (n = 85)	MTX + TNFi (n = 87)	MTX + CTLA-4Ig (n = 92)	MTX + IL-6Ri (n = 83)	P-value
**Age, years**^a^	62 (21–81)	58 (21–79)	58 (18–82)	53 (25–79)	0.29
**Female sex**^b^	58 (68%)	58 (67%)	62 (67%)	57 (69%)	0.99
**BMI, kg/m**^**2**a^	26 (18–43)	25 (19–37)	26 (18–38)	25 (20–43)	0.11
**Current smoker**^b^	12 (14%)	20 (23%)	18 (20%)	22 (27%)	0.22
**Autoantibody status**					0.55
**RF-ACPA-**^b^	11 (13%)	10 (11%)	11 (12%)	4 (5%)	–
**RF+ACPA-**^b^	5 (6%)	6 (7%)	5 (5%)	9 (11%)	–
**RF-ACPA+**^b^	11 (13%)	14 (16%)	12 (13%)	17 (20%)	–
**RF+ACPA+**^b^	57 (67%)	57 (66%)	64 (70%)	53 (64%)	–
**Symptom duration, days**^a,c^	142 (25–813)	144 (41–702)	170 (37–731)	170 (37–691)	0.29
**CDAI**^a^	30.7 (7.8–62.8)	27.9 (8.1–68.7)	29.5 (14–68.4)	26.8 (8.4–55.2)	0.33
**DAS28-CRP**^a^	5.2 (2.6–7.7)	5.1 (2.2–8.3)	5.1 (3.3–7.6)	5.0 (2.7–7.3)	0.21
**DAS28-ESR**^a^	5.6 (3.6–8.2)	5.6 (2.7–8.7)	5.5 (3.7–8.1)	5.3 (2.6–7.9)	0.23
**SJC-66**^a^	13 (2–42)	12 (2–34)	11 (2–41)	10 (1–27)	0.10
**TJC-68**^a^	15 (2–47)	15 (1–47)	14 (0–62)	13 (0–47)	0.55
**CRP, mg/** ^a^	16 (0.5–216)	14 (0.5–180)	11 (0.3–146)	8.4 (0.3–82)	0.19
**ESR, mm/h**^a^	31 (4-108)	32 (4–98)	28 (4–115)	24 (2–84)	0.14
**PGA, mm**^a^	58 (2–87)	57 (13–100)	61 (19–100)	59 (9–100)	0.18

Missing data from one patient regarding BMI, RF, IFN day 1, IFN week 24, CDAI week 24, PGA week 24, and ESR week 24; from two patients regarding CRP day 1 and DAS28-ESR week 24; from three patients regarding CRP week 24; from four patients regarding CDAI day 1 and DAS28-CRP week 24; and from five patients regarding ESR day 1 and DAS28-ESR day 1

*MTX* methotrexate, *TNFi* certolizumab-pegol, *CTLA-4Ig* abatacept, *IL-6Ri* tocilizumab, *BMI* body mass index, *RF* rheumatoid factor, *ACPA* anti-citrullinated protein antibodies, *CDAI* clinical disease activity index, *DAS28* disease activity score 28 joints, *SJC-66* swollen joint count, 66 joints, *TJC-68* tender joint count, 68 joints, *CRP* C-reactive protein, *ESR* erythrocyte sedimentation rate, *PGA* patient global assessment

^a^Median (range), Kruskal-Wallis followed by Dunn’s multiple comparison test

^b^n (%), Fisher’s exact test

^c^Retrospective patient-reported joint pain before RA diagnosis

### IFNα protein positivity is associated with double-positivity for RF and ACPA

To determine the demographic and clinical characteristics of the IFNα protein-positive subgroup, we compared patients who were positive or negative for IFNα protein at baseline. IFNα protein positivity was associated with double-positivity for RF and ACPA, and of IFNα-positive patients, 92% were double-positive for RF and ACPA compared to 57% of IFNα-negative patients. In contrast, only 3% of IFNα-positive patients were double-negative, and only 4% were positive for either RF or ACPA compared to 13% and 29% of IFNα-negative patients, respectively (Table 2 and Additional Figure 1). Baseline IFNα protein positivity was not associated with age, sex, or BMI, and not with disease activity measures at baseline or 24 weeks after treatment initiation. Similar results were obtained when LLOD was used as a cut-off for IFNα positivity (Additional Table 1). When double-positive patients were divided into IFNα-positive and IFNα-negative patients, no significant differences in CDAI day 1 or week 24 (p = 0.07 and p = 0.45 respectively) or DAS28-ESR day 1 or week 24 (p = 0.28 and p = 0.79 respectively) were found.

**Table 2:** Demographic and clinical characteristics of IFNα-positive and IFNα-negative patients

N = 346	IFNα-negative (n = 255)	IFNα-positive^a^ (n = 91)	p-value
**Age, years**^b^	58 (18–81)	58 (21–82)	0.53
**Female sex**^c^	170 (67%)	64 (70%)	0.60
**BMI, kg/m**^**2**b^	25 (18–43)	26 (19–43)	0.22
**Current smoker**^c^	46 (18%)	25 (27%)	0.07
**Autoantibody status**^c^			**< 0.0001**
RF-ACPA-	33 (13%)	3 (3%)	**p < 0.05**^**d**^
RF+ACPA-	23 (9%)	2 (2%)	**ns**
RF-ACPA+	52 (20%)	2 (2%)	**p < 0.05**^**d**^
RF+ACPA+	146 (57%)	84 (92%)	**p < 0.05**^**d**^
**Disease activity day 1**^b^			
CDAI	27.8 (7.8–68.7)	28.6 (10.1–68.4)	0.13
DAS28-CRP	5.1 (2.2–8.3)	5.1 (3.3–7.7)	0.38
DAS28-ESR	5.5 (2.6–8.7)	5.5 (3.3–8.2)	0.43
SJC-66	11 (1–42)	11 (2–38)	0.68
TJC-68	13 (0–49)	16 (2–62)	0.16
CRP, mg/L	14 (0.3–216)	8 (0.5–190)	0.16
ESR, mm/h	28 (2–115)	28 (4–108)	0.26
PGA, mm	59 (2–100)	56 (22–100)	0.59
**Disease activity week 24**^b^			
CDAI	3.4 (0–28.3)	3.5 (0–26.6)	0.47
DAS28-CRP	2.0 (1.1–4.8)	2.0 (1.0–5.0)	0.82
DAS28-ESR	2.3 (0–6.0)	2.2 (0–5.8)	0.91
SJC-66	0 (0–9)	0 (0–7)	0.88
TJC-68	1 (0–37)	2 (0–41)	0.22
CRP, mg/L	1 (0–39)	1 (0.1–15)	0.86
ESR, mm/h	8 (1–78)	8 (1–48)	0.52
PGA, mm	11 (0–78)	14 (0–92)	0.40

*BMI* body mass index, *RF* rheumatoid factor, *ACPA* anti-citrullinated protein antibodies, *CDAI* clinical disease activity index, *DAS28* disease activity score 28 joints, *SJC-66* swollen joint count, 66 joints, *TJC-68* tender joint count, 68 joints, *CRP* C-reactive protein, *ESR* erythrocyte sedimentation rate, P*GA* patient global assessment

^a^IFNα positivity defined as IFNα protein level above 136 fg/mL

^b^Median (range), Mann-Whitney U-test

^c^n (%), Fisher’s exact test

^d^p < 0.05 after post hoc step-down Bonferroni-Holm correction for multiple testing

To evaluate whether the association between IFNα and double-positivity for RF and ACPA was due to demographic or clinical characteristics, multivariable logistic regression analysis was performed (Table 3). Double-positivity for RF and ACPA was associated with IFNα protein positivity and increased the odds ratio of IFNα protein positivity ninefold at baseline and fivefold at week 24 when adjusting for current smoking, CDAI, and CRP. Current smoking independently doubled the odds ratio of IFNα protein positivity at week 24 but neither CDAI nor CRP affected the odds ratio. Taken together, baseline IFNα protein positivity was independently associated with double-positivity for RF and ACPA and smoking but not with disease activity in early RA.

**Table 3:** Factors associated with IFNα positivity at day 1 and week 24

	OR for IFNα positivity at day 1^a^	95% CI	OR for IFNα positivity at week 24^a^	95% CI
**RF+ACPA+**^b^	8.92	**4.21–22.04**	5.24	**2.02–17.95**
**Current smoker**^b^	1.70	0.91–3.15	2.18	**1.01–4.56**
**CDAI day 1**^c^	1.02	1.00–1.04	1.03	1.00–1.06
**CRP day 1**^d^	1.00	0.99–1.01	1.00	0.98–1.01

Multivariable logistic regression with IFNα positivity at day 1 and week 24 as the dependent variable. At day 1, IFNα-positive (n = 91) and IFNα-negative (n = 255). At week 24, IFNα-positive (n = 41) and IFNα-negative (n = 305)

*RF* rheumatoid factor, *ACPA* anti-citrullinated protein antibodies, *CDAI* clinical disease activity index

^a^IFNα positivity defined as IFNα protein level above 136 fg/mL

^b^Yes versus no

^c^Per point increase

^d^Per 1 mg/L increase

### IFNα plasma protein levels decrease to a similar extent in all treatment arms

**Fig. 2:**
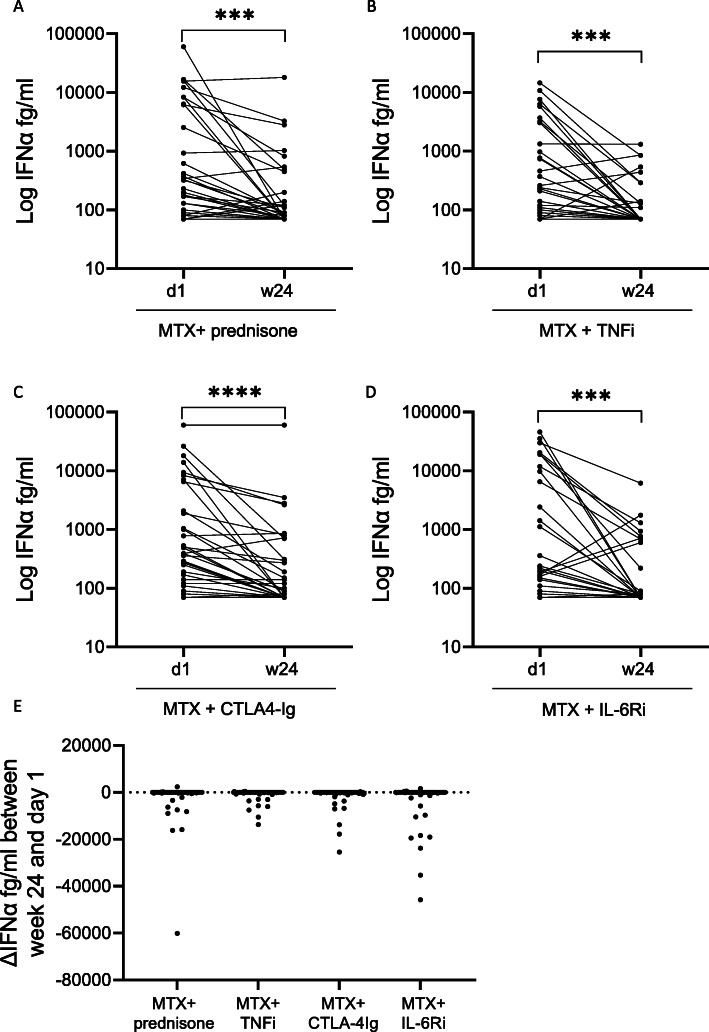
IFNα protein levels are reduced after treatment initiation with conventional and biologic treatment strategies. IFNα protein levels in plasma from patients with early RA before (d1) and 24 weeks after treatment initiation (w24) with **A** methotrexate + prednisone (n = 85), **B** methotrexate + TNFi (n = 87), **C** methotrexate + CTLA-4Ig (n = 91), and **D** methotrexate + IL-6Ri (n = 82). Wilcoxon matched-pairs signed rank test. **E** Absolute difference in IFNα plasma protein levels between week 24 and day 1 in four treatment arms. MTX (methotrexate), TNFi (certolizumab-pegol), CTLA-4Ig (abatacept), and IL-6Ri (tocilizumab). Kruskal-Wallis test followed by Dunn’s multiple comparison test

Next, we investigated the effect of conventional and biologic treatment strategies on IFNα protein levels. IFNα protein levels decreased 24 weeks after treatment initiation in all four treatment arms, and the absolute change in IFNα protein level between day 1 and week 24 did not differ between the treatment arms (Fig. 2A–E).

### Baseline IFNα protein levels do not predict remission at week 24

**Fig. 3:**
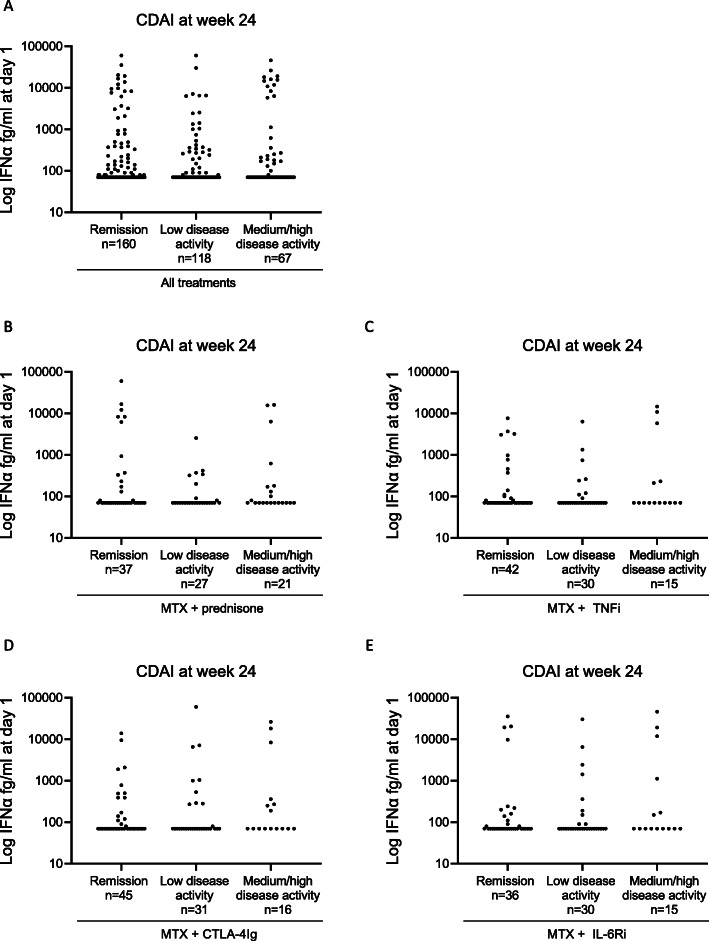
Baseline IFNα protein levels do not predict remission after treatment. Baseline IFNα protein levels in plasma from patients with early RA, stratified according to CDAI 24 weeks after treatment initiation; in remission (CDAI 0–2.8), low disease activity (CDAI 2.9–10.0), and moderate/high disease activity (CDAI 10.1–76.0) with **A** all treatments, **B** methotrexate + prednisone, **C** methotrexate + TNFi, **D** methotrexate + CTLA-4Ig, and **E** methotrexate + IL-6Ri. MTX (methotrexate), TNFi (certolizumab-pegol), CTLA-4Ig (abatacept), IL-6Ri (tocilizumab). Kruskal-Wallis test followed by Dunn’s multiple comparison test

To evaluate IFNα protein in plasma as a biomarker for remission in early RA, we compared baseline IFNα protein levels in patients who achieved CDAI remission at week 24 versus those with low or moderate/high disease activity. Baseline IFNα protein level did not differ according to remission status in the whole group or in any of the treatment arms (Fig. 3A–E). Similar results were obtained when we compared patients who achieved DAS28-ESR remission to those with low or moderate/high disease activity (Additional Figure 2A-E).

To ensure that a potential association between IFNα and remission status was not confounded by factors associated with IFNα, we added IFNα protein positivity, current smoking, and double-positivity for RF and ACPA to a logistic regression model. After adjustment for current smoking and double-positivity, baseline IFNα protein positivity was still not significantly associated with CDAI (OR 0.79, 95% CI 0.47–1.32) or DAS28-ESR (OR 0.64, 95% CI 0.37–1.09) remission at week 24. In addition, in the 127 patients with IFNα levels above LLOD, the baseline IFNα protein level did not correlate with CDAI or DAS28-ESR at baseline, CDAI or DAS28-ESR at week 24, or absolute change in CDAI or DAS28-ESR from baseline until week 24 (Additional Figure 3). Thus, the baseline protein level of IFNα did not predict remission 24 weeks after treatment initiation in patients with early RA.

## Discussion

The expression of IRG is upregulated in a subgroup of patients with RA, but IFNα protein levels have not previously been determined in RA. We demonstrate for the first time that IFNα protein positivity is present in a subgroup of patients with untreated early RA. IFNα protein positivity was strongly associated with double-positivity for RF and ACPA but not with disease activity. Treatment with both conventional and biologic DMARDs led to decreased levels of IFNα protein, but the absolute change did not differ between the treatment arms. Pre-treatment levels of IFNα protein did not predict remission at week 24.

Previously, gene variants of interferon regulatory factor-5 (IRF-5) were shown to be associated with seronegative RA [26, 27], leading to the notion that the type I IFN pathway may be more important in autoantibody-negative patients. Here, we show that double-positivity for RF and ACPA is associated with increased risk for IFNα protein positivity, while single-positivity and double-negativity are related to IFNα negativity. One explanation could be that RF and ACPA in combination might induce a more potent stimulation of IFNα protein production. Indeed, double-positive patients with RA exhibit higher levels of the proinflammatory cytokines TNF, IL-6, and IL-1β than single-positive patients [28]. However, it is also possible that IFNα can induce the production of RF and ACPA. IFNα stimulates B cell activating factor [29, 30], plasma cell differentiation, and antibody secretion [31]. Thus, IFNα may stimulate RF and ACPA autoantibody production, which form immune complexes that may in turn stimulate plasmacytoid dendritic cells to produce IFNα protein.

The cut-off for IFNα positivity was 136 fg/mL, based on 3 SD above mean level for 68 healthy blood donors [21]. We obtained similar results when using LLOD as the cut-off. When we measured IFNα protein in 27 healthy controls, all had values below LLOD. Using the same cut-off, 52% of patients with SLE were IFNα-positive [21] compared to 26% of early RA patients in the present study. This is in line with previous results, where lower IRG expression has been seen in RA compared to SLE [9, 32]. Nucleic acids stimulate IFNα protein production from plasmacytoid dendritic cells, and elevated IFNα protein levels in SLE are associated with the presence of autoantibodies against DNA, ribonucleoprotein, and the RNA-binding Smith antigen [21]. Thus, an explanation for the larger proportion of IFNα-positive patients in SLE relative to RA may be that autoantibodies in SLE target endogenous nucleic acids that may be more potent than RF and ACPA in stimulating IFNα protein production. Besides the presence of autoantibodies, SLE and RA share several pathological features including joint pain, fatigue, and a female predisposition, and the diseases may overlap. Therefore, the shared overexpression of IFNα in subgroups of patients with SLE and RA may contribute to the similarities between the diseases. Since the IFNα/β receptor inhibitor anifrolumab suggested improvements to primary or secondary outcomes in SLE [33, 34], it will be interesting to see whether RA patients with high IFNα protein level may benefit from this medication.

Increased IRG expression is evident in early and established RA. Although the definition varies, elevated IRG expression was described in 42–61% of patients with early RA [10, 11] and 21–57% of patients with established RA [9, 11, 12, 35–37]. While its effect on remission is unknown, IRG expression has been associated with disease activity in early RA. Elevated baseline IRG expression associated with increased DAS28 6 months after treatment initiation with MTX and glucocorticoid [5] as well as MTX, intramuscular glucocorticoid, and/or hydroxychloroquine [11]. However, another study found no association to disease activity 6 months after treatment initiation with MTX, prednisolone, and/or sulfasalazine [38]. In the present study, IFNα protein positivity was not related to disease activity or remission 6 months after initiation of conventional or biologic treatment.

IRG expression has been suggested as a predictive biomarker for the response to biologic therapies. High or increasing IRG expression associated with poor response to anti-TNF treatment [12, 13] although one study reported association with good response [14] and one saw no association [6]. Whether IRG expression predicts response to CTLA-4Ig has not been studied, but high IRG expression was also suggested to predict a good response to anti-IL-6Ri treatment [15]. On a protein level, however, we found that IFNα protein levels decreased irrespective of treatment, and the baseline IFNα protein level did not differ according to remission status in any of the treatment arms. The B-cell depleting agent rituximab was not included as one of the treatment arms, since it is not recommended as the first biological treatment in RA by Swedish or European guidelines. Given the association to autoantibody positivity, it would be of interest to evaluate IFNα protein as a biomarker for treatment effect by rituximab. Indeed, low pre-treatment IRG expression was shown to predict good response to rituximab [16–19]. IFNα stimulates B cell survival, and the repopulation of depleted B-cells may be accelerated in patients with high IRG expression. In addition, since IFNα exerts its effect through the JAK-STAT pathway, it is relevant to examine whether IFNα protein level may predict treatment effect to JAK inhibitors in early RA.

This study uses data and plasma samples from the investigator-initiated NORD-STAR study in early untreated RA, and the clinical trial design with randomization to four different treatment arms is a major strength. In addition, previous studies have used proxy markers such as IRG expression to evaluate the role of IFNα in RA, while we were able to sensitively measure the levels of IFNα protein in plasma. However, one limitation is that we do not have data for both IRG expression and IFNα plasma levels. Further, the titers of RF and ACPA were measured at different laboratories, which precludes the analysis of autoantibody levels in relation to IFNα protein levels.

## Conclusions

In conclusion, IFNα protein positivity was present in a subgroup of patients with early untreated RA and associated with double-positivity for RF and ACPA, but not with disease activity, and did not predict remission 24 weeks after treatment initiation. The association between IFNα and double-positivity for autoantibodies warrants further investigation regarding the role of IFNα in the pathogenesis of early RA. For example, measurement of IFNα protein in synovial fluid would be of value to elucidate the role of IFNα in the local inflammation of the joint.

## Supplementary Information

**Additional file 1:.** Figure S1. IFNα protein positivity is associated with double-positivity for RF and ACPA. RF/ACPA status in patients who are IFNα positive and IFNα negative at baseline.

**Additional file 2:.** Figure S2. Baseline IFNα protein levels do not predict remission in any of the treatment arms. Baseline IFNα protein levels in plasma from patients with early RA stratified according to DAS28-ESR 24 weeks after treatment initiation; in remission (DAS28-ESR < 2.6), low disease activity (2.6 < DAS28-ESR ≤ 3.2) or moderate/high disease activity (DAS28-ESR > 3.2) with A) all treatments, B) methotrexate + prednisone, C) methotrexate + TNFi, D) methotrexate + CTLA-4Ig and E) methotrexate + IL-6Ri. MTX (methotrexate), TNFi (certolizumab-pegol), CTLA-4Ig (abatacept), IL-6Ri (tocilizumab). Kruskal-Wallis test followed by Dunn’s multiple comparison test.

**Additional file 3:.** Figure S3. IFNα protein levels at baseline do not correlate with CDAI or DAS28-ESR. Correlation between IFNα protein level in patients with levels above detection limit at day 1 and A) CDAI day 1, B) CDAI week 24, C) absolute difference in CDAI between week 24 and day 1, D) DAS28-ESR day 1, E) DAS28-ESR week 24 and F) absolute difference in DAS28-ESR between week 24 and day 1. Spearman rank correlation coefficient.

**Additional file 4:.** Table S1 Demographic and clinical characteristics of patients with IFNα below or above lowest limit of detection

## Data Availability

All data relevant to the study is included in the article or uploaded as supplementary information. Data are available upon reasonable request.

## Abbreviations

ACPA: Anti-citrullinated protein antibodies
ACR: American College of Rheumatology
BMI: Body mass index
CDAI: Clinical disease activity index
CRP: C-reactive protein
CTLA-4Ig: Cytotoxic T-lymphocyte-associated molecule-4 immunoglobulin
DAS28: Disease activity score, 28 joints
DMARD: Disease-modifying anti-rheumatic drug
ESR: Erythrocyte sedimentation rate
EULAR: European League Against Rheumatism
IFN: Interferon
IL-6Ri: Interleukin-6 receptor inhibitors
IRF: Interferon regulatory factor
IRG: Type I IFN responsive genes
LLOD: Lowest limit of detection
MTX: Methotrexate
NORD-STAR: Nordic rheumatic diseases strategy trials and registries
PGA: Patient global assessment
RA: Rheumatoid arthritis
RF: Rheumatoid factor
Simoa: Single molecular array
SJC-66: Swollen joint count in 66 joints
SLE: Systemic lupus erythematosus
TJC-68: Tender joint count in 68 joints
TNFi: Tumor necrosis factor inhibitors
