# Supplementary figures and images for: Plasma interferon-alpha is associated with double-positivity for autoantibodies but is not a predictor of remission in early rheumatoid arthritis—a spin-off study of the NORD-STAR randomized clinical trial
Source: Arthritis Res Ther. 2021 Jul 13;23:189. doi: 10.1186/s13075-021-02556-1 (PMC8278690; doi:10.1186/s13075-021-02556-1)

## Slide 1
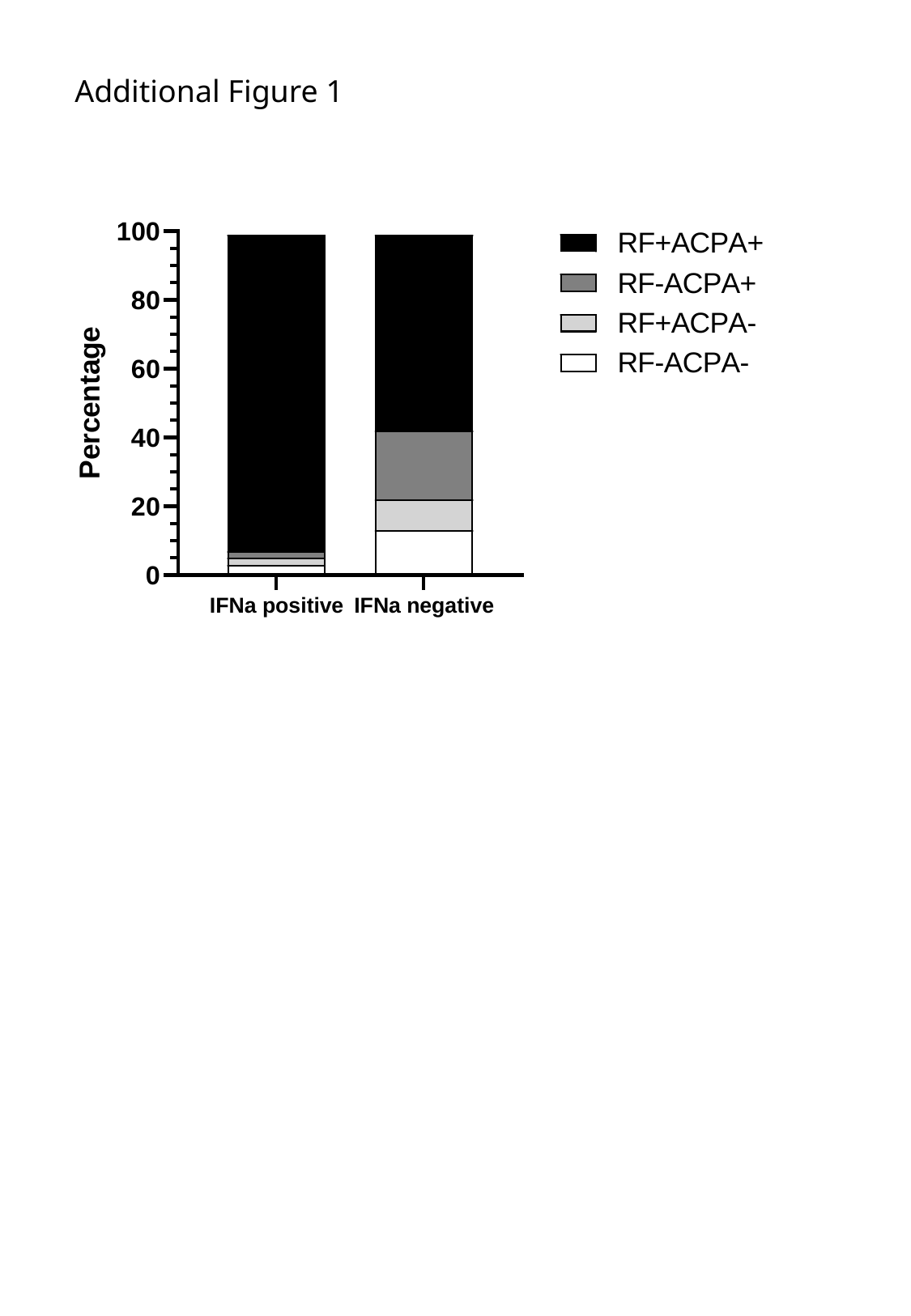

# Additional Figure 1

Supplement: Supplementary file 1 — Additional file 1:. Figure S1. IFNα protein positivity is associated with double-positivity for RF and ACPA. RF/ACPA status in patients who are IFNα positive and IFNα negative at baseline. [file 13075_2021_2556_MOESM1_ESM.pptx]

## Slide 1
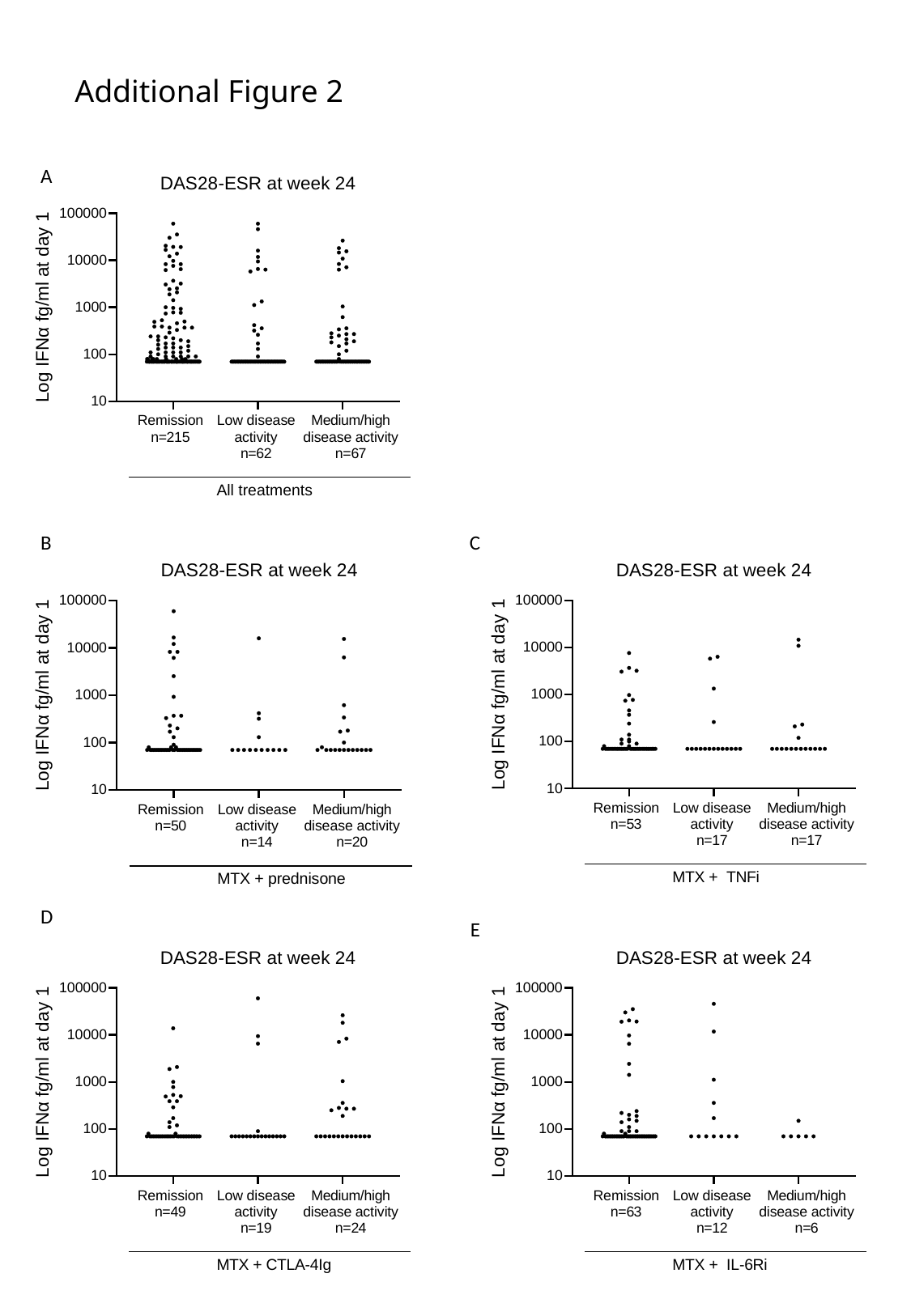

# Additional Figure 2
A
B
C
D
E

Supplement: Supplementary file 2 — Additional file 2:. Figure S2. Baseline IFNα protein levels do not predict remission in any of the treatment arms. Baseline IFNα protein levels in plasma from patients with early RA stratified according to DAS28-ESR 24 weeks after treatment initiation; in remission (DAS28-ESR < 2.6), low disease activity (2.6 < DAS28-ESR ≤ 3.2) or moderate/high disease activity (DAS28-ESR > 3.2) with A) all treatments, B) methotrexate + prednisone, C) methotrexate + TNFi, D) methotrexate + CTLA-4Ig and E) methotrexate + IL-6Ri. MTX (methotrexate), TNFi (certolizumab-pegol), CTLA-4Ig (abatacept), IL-6Ri (tocilizumab). Kruskal-Wallis test followed by Dunn’s multiple comparison test. [file 13075_2021_2556_MOESM2_ESM.pptx]

## Slide 1
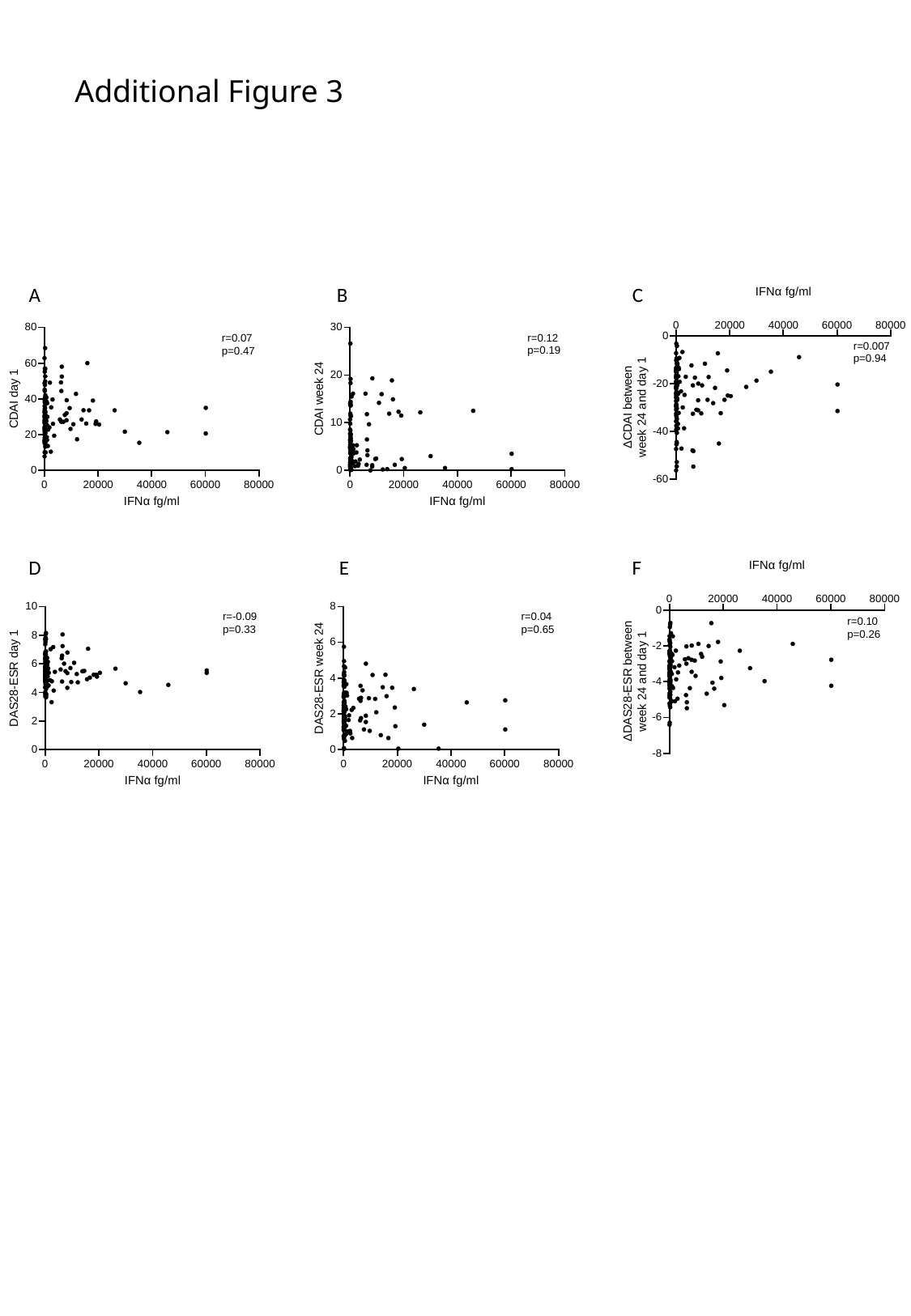

# Additional Figure 3
A
B
C
D
E
F

Supplement: Supplementary file 3 — Additional file 3:. Figure S3. IFNα protein levels at baseline do not correlate with CDAI or DAS28-ESR. Correlation between IFNα protein level in patients with levels above detection limit at day 1 and A) CDAI day 1, B) CDAI week 24, C) absolute difference in CDAI between week 24 and day 1, D) DAS28-ESR day 1, E) DAS28-ESR week 24 and F) absolute difference in DAS28-ESR between week 24 and day 1. Spearman rank correlation coefficient. [file 13075_2021_2556_MOESM3_ESM.pptx]
