# Supplementary material for: Plasma interferon-alpha is associated with double-positivity for autoantibodies but is not a predictor of remission in early rheumatoid arthritis—a spin-off study of the NORD-STAR randomized clinical trial
Source: Arthritis Res Ther. 2021 Jul 13;23:189. doi: 10.1186/s13075-021-02556-1 (PMC8278690; doi:10.1186/s13075-021-02556-1)
Supplement: Supplementary file 4 — Additional file 4:. Table S1 Demographic and clinical characteristics of patients with IFNα below or above lowest limit of detection [file 13075_2021_2556_MOESM4_ESM.docx]

### **Additional Table 1. Demographic and clinical characteristics of patients with IFNα below or above lowest limit of detection**

| **N=346** | | **IFNα <LLOD**  **n=219** | **IFNα ≥LLOD**  **n=127** | **p-value** |
| --- | --- | --- | --- | --- |
| **Age, years** ^a^ | | 59 (18-81) | 57 (21-82) | 0.88 |
| **Female sex** ^b^ | | 146 (67%) | 88 (69%) | 0.64 |
| **BMI, kg/m^2^** ^a^ | | 25 (18-43) | 26 (19-43) | 0.67 |
| **Current smoker** ^b^ | | 42 (19%) | 29 (23%) | 0.41 |
| **Autoantibody status** ^b^ | |  |  | **<0.0001** |
|  | RF-ACPA- | 33 (15%) | 3 (2%) | **p<0.05^d^** |
|  | RF+ACPA- | 19 (9%) | 6 (5%) | ns |
|  | RF-ACPA+ | 49 (22%) | 5 (4%) | **p<0.05^d^** |
|  | RF+ACPA+ | 117 (53%) | 113 (89%) | **p<0.05^d^** |
| **Disease activity day 1** ^a^ | |  |  |  |
|  | CDAI | 27.7 (8.1-68.7) | 28.5 (7.8-68.4) | 0.30 |
|  | DAS28-CRP | 5.1 (2.2-8.3) | 5.1 (2.6-7.7) | 0.52 |
|  | DAS28-ESR | 5.5 (2.6-8.7) | 5.5 (3.3-8.2) | 0.55 |
|  | SJC-66 | 12 (1-42) | 10 (2-38) | 0.46 |
|  | TJC-68 | 14 (0-49) | 15 (2-62) | 0.58 |
|  | CRP, mg/L | 14 (0.3-216) | 9 (0.5-190) | 0.19 |
|  | ESR, mm/h | 28 (2-102) | 29 (4-115) | 0.48 |
|  | PGA, mm | 59 (2-100) | 58 (12-100) | 0.42 |
| **Disease activity week 24** ^a^ | |  |  |  |
|  | CDAI | 3.7 (0-28.3) | 3.2 (0-26.6) | 0.72 |
|  | DAS28-CRP | 2.1 (1.1-4.8) | 1.9 (1.0-5.0) | 0.34 |
|  | DAS28-ESR | 2.3 (0-6.0) | 2.1 (0-5.8) | 0.23 |
|  | SJC-66 | 0 (0-9) | 0 (0-7) | 0.13 |
|  | TJC-68 | 2 (0-37) | 1 (0-41) | 0.82 |
|  | CRP, mg/L | 1 (0.1-33) | 1 (0.1-39) | 0.78 |
|  | ESR, mm/h | 8 (1-72) | 8 (1-78) | 0.49 |
|  | PGA, mm | 11 (0-78) | 14 (0-92) | 0.57 |

Lowest limit of detection (LLOD, 70 fg/ml), body mass index (BMI), rheumatoid factor (RF), anti-citrullinated protein antibodies (ACPA), clinical disease activity index (CDAI), disease activity score 28 joints (DAS28), swollen joint count 66 joints (SJC-66), tender joint count 68 joints (TJC-68), C-reactive protein (CRP), erythrocyte sedimentation rate (ESR), patient global assessment (PGA).

^a^ Median (range), Mann Whitney U-test.

^b^ n (%), Fisher’s exact test.

^d^ p<0.05 after post-hoc step-down Bonferroni-Holm correction for multiple testing
